# Rapid Development and Use of a Nationwide Training Program for Cholera Management, Haiti, 2010

**DOI:** 10.3201/eid1711.110857

**Published:** 2011-11

**Authors:** Robert V. Tauxe, Michael Lynch, Yves Lambert, Jeremy Sobel, Jean W. Domerçant, Azharul Khan

**Affiliations:** Centers for Disease Control and Prevention, Atlanta, Georgia, USA (R. Tauxe, M. Lynch, J. Sobel); University of Haiti School of Medicine, Port-au-Prince, Haiti (Y. Lambert); International Training and Education Center for HIV/AIDS, Port-au-Prince (Y. Lambert); Centers for Disease Control and Prevention, Port-au-Prince (J.W. Domerçant); International Centre for Diarrheal Disease Research, Dhaka, Bangladesh (A. Khan)

**Keywords:** enteric infections, waterborne infections, Cholera, clinical training, Haiti, epidemic response, online resources, train-the-trainer, training of trainers, disease management

## Abstract

When epidemic cholera appeared in Haiti in October 2010, the medical community there had virtually no experience with the disease and needed rapid training as the epidemic spread throughout the country. We developed a set of training materials specific to Haiti and launched a cascading training effort. Through a training-of-trainers course in November 14–15, 2010, and department-level training conducted in French and Creole over the following 3 weeks, 521 persons were trained and equipped to further train staff at the institutions where they worked. After the training, the hospitalized cholera patients’ case-fatality rate dropped from 4% to <2% by mid-December and was <1% by January 2011. Continuing in-service training, monitoring and evaluation, and integration of cholera management into regular clinical training will help sustain this success.

## Abstract 2

Rapid training of health care staff was followed by lower death rates.

When toxigenic *Vibrio cholerae* O1 was identified in Haiti on October 21, 2010, it was soon apparent that the epidemic would be severe and clinical training needs great (*1*). Epidemic cholera had never been reported from Haiti, and the clinical community there had virtually no experience with the disease. By November 1, a total of 6,422 hospitalized patients with cholera were reported from 5 of the 10 departments of Haiti (*2*). Of these patients, 244 had died, resulting in a hospitalized case-fatality rate (CFR) of 3.8%. The CFR for untreated clinical cholera is >20% (*3*), but with access to care and aggressive appropriate volume replacement, it can be reduced to <1% (*4*). In the 1991 Latin American cholera epidemic, transmission was sustained in countries with better water and sanitation and lower infant mortality rates than Haiti, suggesting that the risk for continued transmission in Haiti would be high (*5*,*6*). The unfortunate concurrence in Haiti of an earthquake-ravaged infrastructure; long-standing deficiencies in water, sanitation and transportation; and the limited number of health professionals and their lack of experience with cholera treatment all suggested that further spread was not only likely but would have severe clinical consequences.

In collaboration with the US Centers for Disease Control and Prevention (CDC), the Haiti Ministère de Santé Publique et de la Population (MSPP) immediately launched a cascading approach to train clinical care providers, using the training-of-trainers approach that has been integral to laboratory and programmatic capacity building in the President’s Emergency Program for AIDS Relief (PEPFAR) in many countries (*7*,*8*). Training in cholera treatment supported the MSPP in reinforcing cholera treatment in existing care facilities and in setting up new centers. Many nongovernmental organizations (NGOs) operate in the Haitian health sector, so this training needed to address a range of public and NGO health care providers with varying skills.

After we developed a package of training materials, clinical training occurred in 3 stages. First, a group of master trainers were trained in Port-au-Prince. They then formed 5 teams, each responsible for training health facility staff in 2 departments in the next 2 weeks, supported by department health authorities. This training was followed by on-site training at health facilities. The training package was also provided to primary PEPFAR NGO partners (e.g., Partners in Health, Haitian Group for the Study of Kaposi’s Sarcoma and Opportunistic Infections, and Catholic Relief Services) for use in their training sessions and was made available by website to all NGOs in Haiti.

To monitor effectiveness of treatment in the short term, we planned to use the hospitalized CFRs from ongoing national surveillance collected by MSPP (*2*). This surveillance provided rapid and consistent information from each department; we thought the hospitalized CFRs would be more complete and would better reflect the clinical treatment outcomes than the overall CFR. We also planned to conduct evaluation of care in health facilities and cholera treatment centers (CTCs) throughout Haiti to identify areas for long-term improvement in diarrheal disease management.

## Developing Training Materials

In the 3 weeks following the first report of cholera, a package of modular training materials was developed that supported varied training needs, including information on the basic management, epidemiology, and prevention of cholera, and instruction relevant for conditions in Haiti. The training included management of temporary CTCs, whether freestanding or within existing health centers. The package also included information for use at the community level on cholera prevention and use of oral rehydration solutions (ORS).

Previously developed materials were updated, combined, and translated into French and Creole. Our work was informed by 1) pamphlets and videos developed by CDC with the Pan American Health Organization (PAHO) in response to the 1991 Latin American cholera epidemic (*9*); 2) the Cholera Outbreak Training and Shigellosis Program of the International Centre for Diarrheal Disease Research, Bangladesh (ICDDR,B), a package that includes a reference manual, presentations, and pocket information cards specific to each work role (*10*) and that was used in Pakistan earlier in 2010, when cholera appeared after a major flood disaster (*11*); 3) short videos produced by ICDDR,B that illustrated setting up CTCs and treating cholera patients in challenging circumstances (*12*,*13*); 4) standard cholera reference materials developed by the World Health Organization (*14*,*15*); and 5) guidelines of the Médecins Sans Frontières (*16*).

We sought input from other groups with cholera expertise. We reviewed our antimicrobial drug recommendations on the basis of susceptibility testing of Haitian epidemic *V. cholerae* isolates (*17*) with PAHO technical experts. We had favored single-dose doxycyline therapy for children, because the risk of dental staining following a single dose seemed far less than the benefit of treating cholera. However, PAHO experts voiced concern that this recommendation might alter routine prescription practices in the region, leading to frequent treatment of childhood diarrhea with doxycycline. Therefore, other effective treatments for pediatric patients were recommended according to resistance of the pathogen strain and availability of antimicrobial agents. An ICDDR,B expert in cholera clinical management and training joined the development team and participated in the training in Haiti. Médecins Sans Frontières clinicians and logistics experts helped us adapt their materials. Finally, all materials were reviewed and approved by the Haitian MSPP. CDC staff in Haiti worked closely with MSPP to make adjustments to fit the circumstances in Haiti.

Although the primary languages used in preparing materials were French and Creole, some materials were also prepared in English and Spanish for use by those participants whose medical training had been in those languages. The training package was produced as hard copy, placed on thumb drives, and made available on CDC’s website (www.cdc.gov/haiticholera/training/hcp_materials.htm).

## Training of Trainers, Port-au-Prince, November 2010

The goal of this course was to cover the practical essentials of treatment, epidemiology, and prevention of cholera so that those trained could then immediately train health care providers. A group of 33 master trainers was identified, drawn mainly from CDC locally employed staff and PEPFAR partners with experience in adult learning. Other health officials also attended; 45 persons took the training-of-trainers course.

The first day covered basic clinical concepts of toxigenic *V. cholerae* infection, pathophysiology of the disease, clinical assessment and treatment, and prevention measures. Trainers mastered the different levels of dehydration and learned to tailor care, treatment, and support while taking into account the limited infrastructure, human resources, and supplies. They learned the elements of setting up a CTC, disease reporting, and surveillance. Principal instructors included 3 of the authors (R.V.T., Y.L., and A.K.), with organizational support for the training from CDC/Haiti and the International Training and Education Center for Health, Haiti.

On the second day the trainers formed small groups to develop and then themselves present an aspect of care, treatment, support, infection control, or prevention of cholera. A site visit to a nearby CTC provided an opportunity to observe cholera patients, review clinical management of severe and moderate dehydration, and observe the CTC layout and infection control procedures.

## Department Training

By November 15, 2011, MSPP reported confirmed cholera in 7 departments and Port-au-Prince, and a total of 18,383 hospitalizations and 729 hospital–associated deaths had been reported, with a cumulative hospitalized CFR of 4% (*2*). Department-level training was conducted over the next 3 weeks in all 10 departments of the country (Figure).

**Figure:**
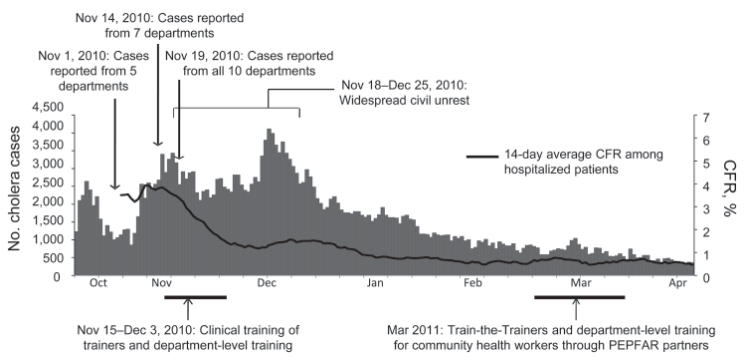
Major events in training, number of cholera cases reported to Ministère de la Santé Publique et de la Population (MSPP) national surveillance by day, and smoothed 14-day case-fatality rate (CFR) for hospitalized calculated from MSPP surveillance data during the cholera epidemic in Haiti, October 20, 2010–April 20, 2011. The first cases were confirmed in Artibonite Department October 21, 2010; by November 19, cholera was reported in all 10 departments in Haiti. PEPFAR, President’s Emergency Program for AIDS Relief.

Each team was assigned 2 departments; equipped with training materials, a projector, and 2 vehicles; and led by CDC regional staff and representatives of the health departments where the training was to be held. Twelve department-level training sessions were conducted, at least 1 in each department. Each team also visited up to 3 functioning CTCs in each department to assess local needs for further training. In departments not yet affected, they visited and assessed facilities proposed as future CTC sites. Critical supplies for first response were provided in some areas to tide centers over while departmental supply logistics were activated.

Nurses, physicians, and pharmacists from all health centers with hospital beds were invited to participate in the department training sessions. These 1-day sessions covered the basic skills needed to care for and treat cholera patients and set up treatment units within their facilities; clarified the need for adequate personnel and supply logistics; and reviewed infection control. The health care providers were also given cards with which to train community health workers on prevention activities, as described by A. Rajasingham et al. (*18*). Personnel in nine departments were trained before civil unrest around the National Election on November 28 complicated travel; by the following week, when department-level training was completed, 521 persons had been trained. One experienced trainer remained in each department to further replicate training and to provide local technical assistance. In each department, further training then began at the health facility level, but the numbers trained were not collected.

Immediately after these sessions, the training teams provided the development team with suggestions for revisions, which were based on questions that arose during the training. The materials were modified to stress even further the primacy of rehydration therapy, to cover the treatment of chronically malnourished patients in more detail, to encourage antimicrobial drug treatment of moderately dehydrated as well as severely dehydrated patients, and to describe more systematically the logistics process for supplies. We also developed a short downloadable synopsis for medical volunteers going to Haiti to staff cholera treatment sites.

Outpatient rehydration and triage of patients with diarrheal illness should reduce the number of cases seeking care at hospitals for severe dehydration. Therefore, community health worker training using another packet of training materials was conducted in early March (*18*).

The training at the department level was enthusiastically received, and trainees reported anecdotally that they would put the knowledge to use immediately. Rapid review in the field of pre-course surveys showed that many trainees entered the training unaware of the basics of cholera treatment but understood the essentials by the end of the course. Regrettably, the assessment forms were then misplaced and were not available for analysis for this synopsis. More objectively, although the number of reported cases increased through December, the CFR rate for hospitalized patients dropped below 2% by mid December and was below 1% by early January (Figure). It has remained there through the end of July, even during a summer increase in cases and even as many NGOs that assisted with the epidemic withdrew. Several factors likely contributed to the decrease in CFR, including expanded support for treatment facilities, improved supply chains, and the growing competence and confidence of caregivers trained in cholera treatment.

## Conclusions

Monitoring and evaluation of the outcomes of training are part of continuous improvement (*19*). Trends in the health outcomes of incidence and CFRs for hospitalized patients provide the most immediate measure of effect and will need continued monitoring. Longer term evaluation and training are being planned now, including assessing the need for refresher and in-service training. In addition to the CFR for hospitalized patients, the long-term success of training can be measured by its sustained influence on the performance of providers at the department or even health care center level. Measurable hallmarks of good clinical management include efficient triage of patients with diarrhea, rapid diagnosis and assessment by physicians, and swift and appropriate treatment by nursing staff. Other performance measures that the ICDDR,B has found useful include comparing the number of persons treated for cholera in a CTC with the volume of intravenous fluids and ORS used at the facility in the same period of time and tracking the average time it takes to discharge patients. Furthermore, assessing logistical plans may help avert shortages of crucial supplies.

Measuring the number of professionals trained, persons reached, commodities distributed, and service points supported can monitor the increase in capacity, but it will also be vital to assess how much difference training makes in practice (*20*,*21*). Not all parts of a training program are equally effective and relevant. Changes may be needed if, for example, the antimicrobial drug resistance of *V. cholerae* O1 changes.

Cholera may persist in Haiti for years, so cholera training needs to be integrated into the curricula of medical, nursing, and pharmacy schools in Haiti. Practical hands-on training in the assessment of dehydration and the use of oral and intravenous rehydration can help trainees transfer new skills to the clinical setting (*22*). One or more ORS treatment centers maintained in academic settings could provide such practical training, which would be of benefit for the treatment of any dehydrating diarrheal illness, so that clinicians and caregivers continue to be well trained and confident in their skills. Such a center could also train clinicians from other countries in the hemisphere, who might otherwise have little chance to become familiar with cholera and its treatment.
